# Supplementary material for: Gene drive and genetic sex conversion in the global agricultural pest Ceratitis capitata
Source: Nat Commun. 2024 Jan 8;15:372. doi: 10.1038/s41467-023-44399-1 (PMC10774415; doi:10.1038/s41467-023-44399-1)
Supplement: Supplementary file 1 — Supplementary Information [file 41467_2023_44399_MOESM1_ESM.pdf]

Figure S1.

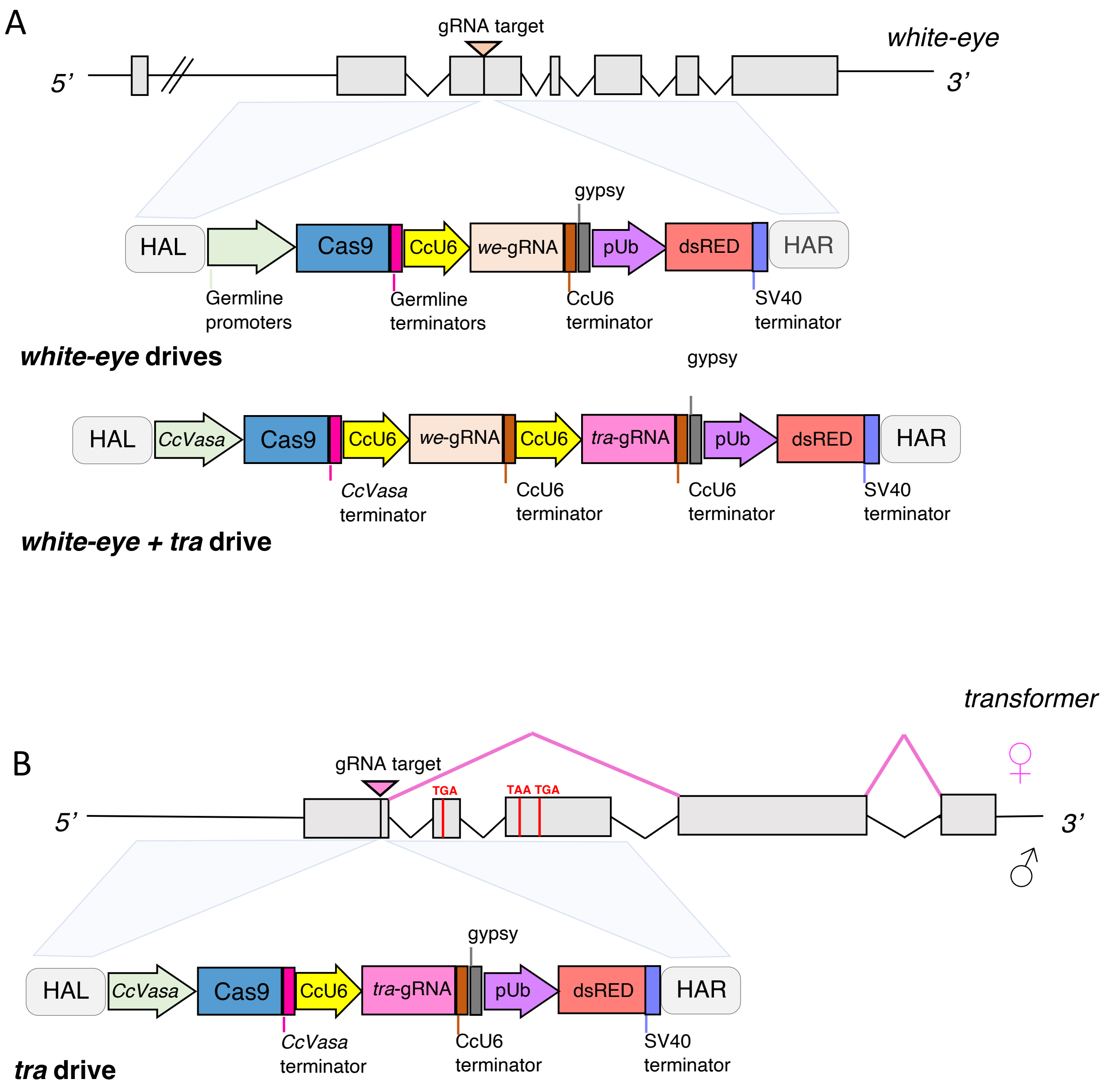

Figure S1. Gene drive constructs and target genes.

Figure S2.

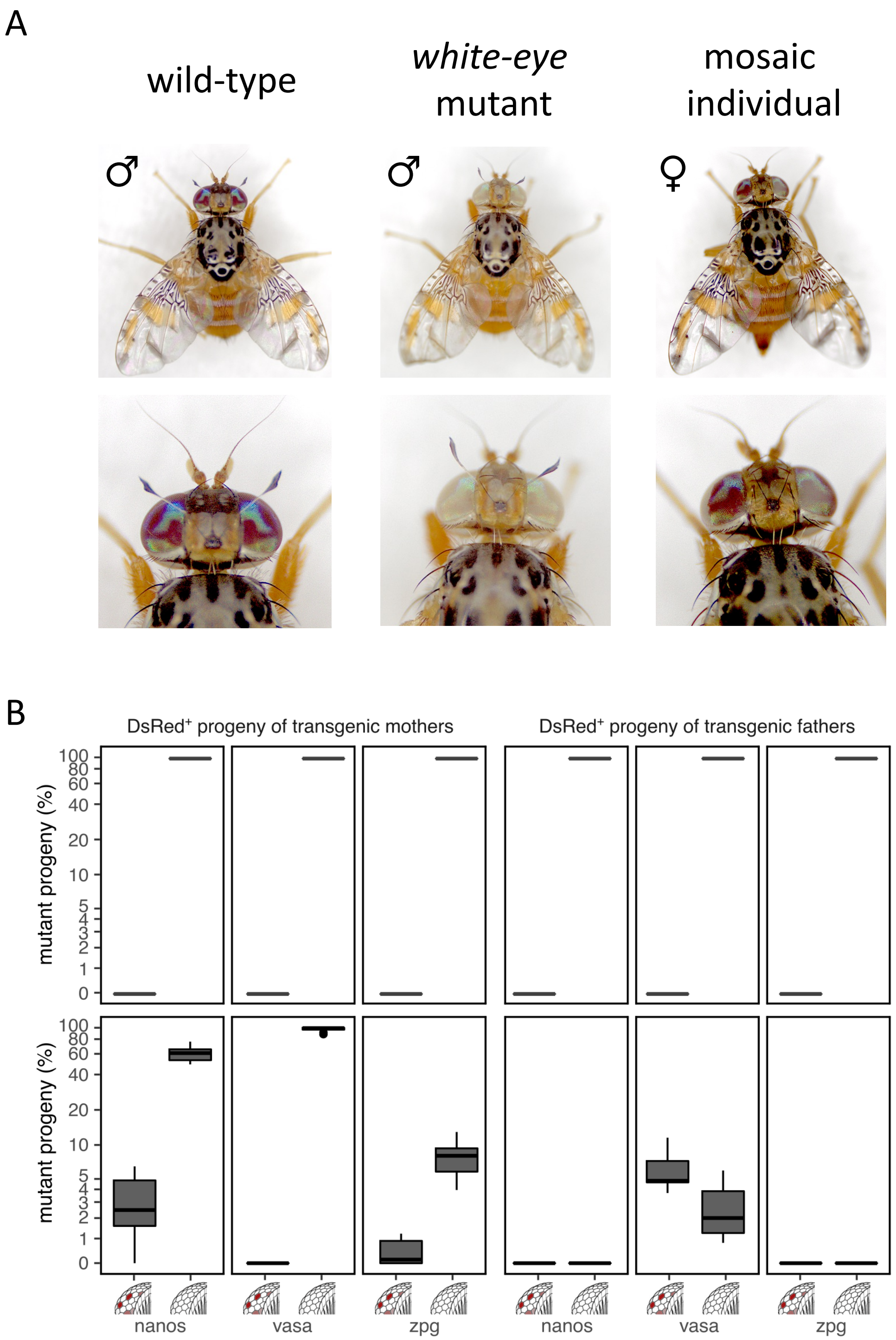

Figure S2. Eye pigmentation in the offspring of *white-eye* drive carriers. A. Eye pigmentation phenotypes of exemplary wild type, *white-eye* mutant and mosaic individuals. **B.** Eye pigmentation phenotypes of the DsRed positive fraction of the progeny of transgenic fathers or mothers carrying the *white-eye* drive constructs with Cas9 being driven by the *nanos*, *vasa* and *zpg* promoters. Shown is the percentage of white eyed and mosaic progeny obtained for each construct with red eyed progeny making up the remainder. Source data are provided as a Source Data file.

Figure S3.

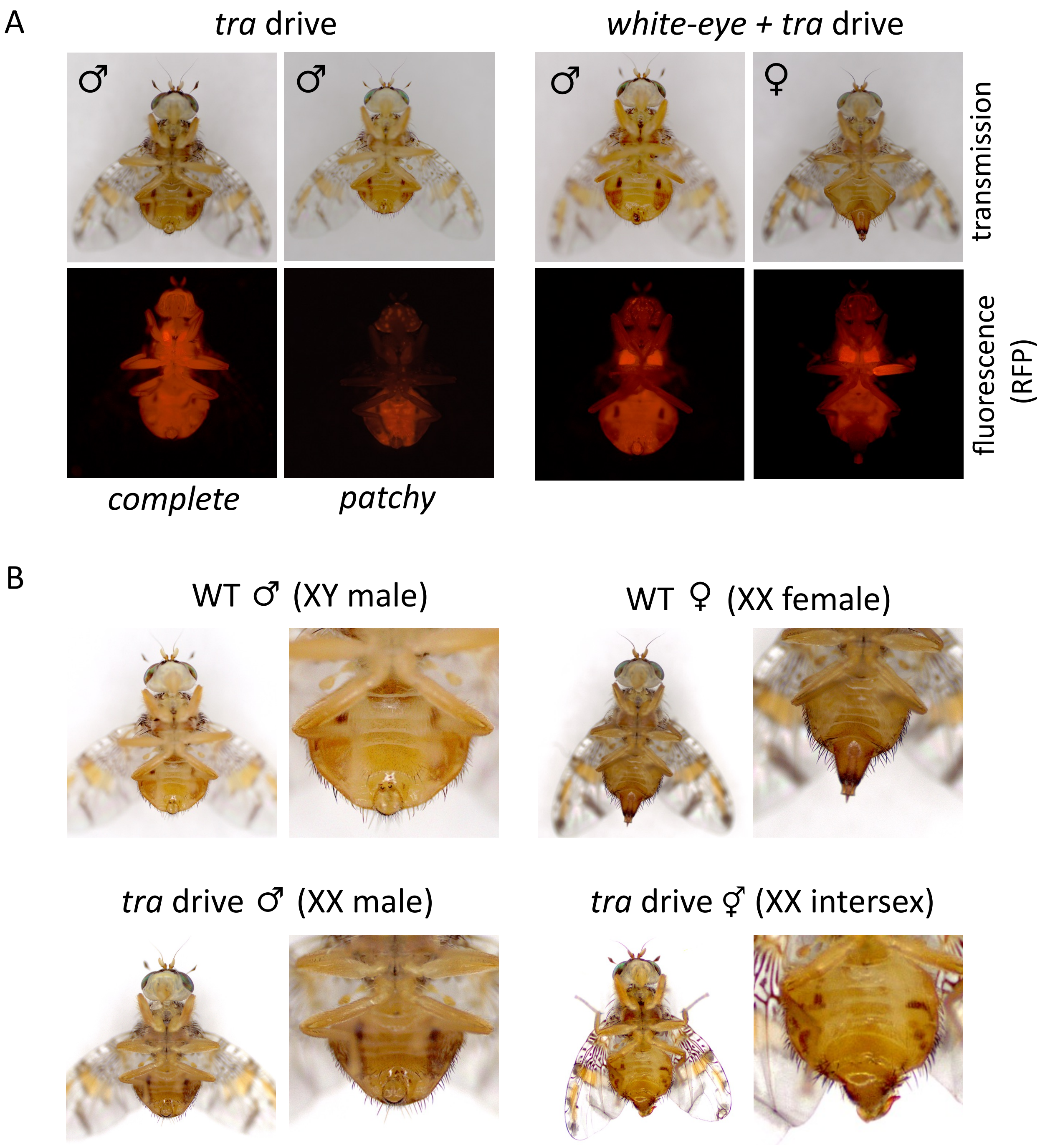

Figure S3. Fluorescence and sexual phenotypes of *tra* drive & *white-eye + tra* drive individuals. A. Transmission and red fluorescence micrographs of *tra* drive (left panels) and *white-eye + tra* drive (right panels) individuals. **B.** Sexual phenotypes of wild type (WT) and *tra* drive individuals (left panels) and close up images of genitalia (right panels).

Figure S4.

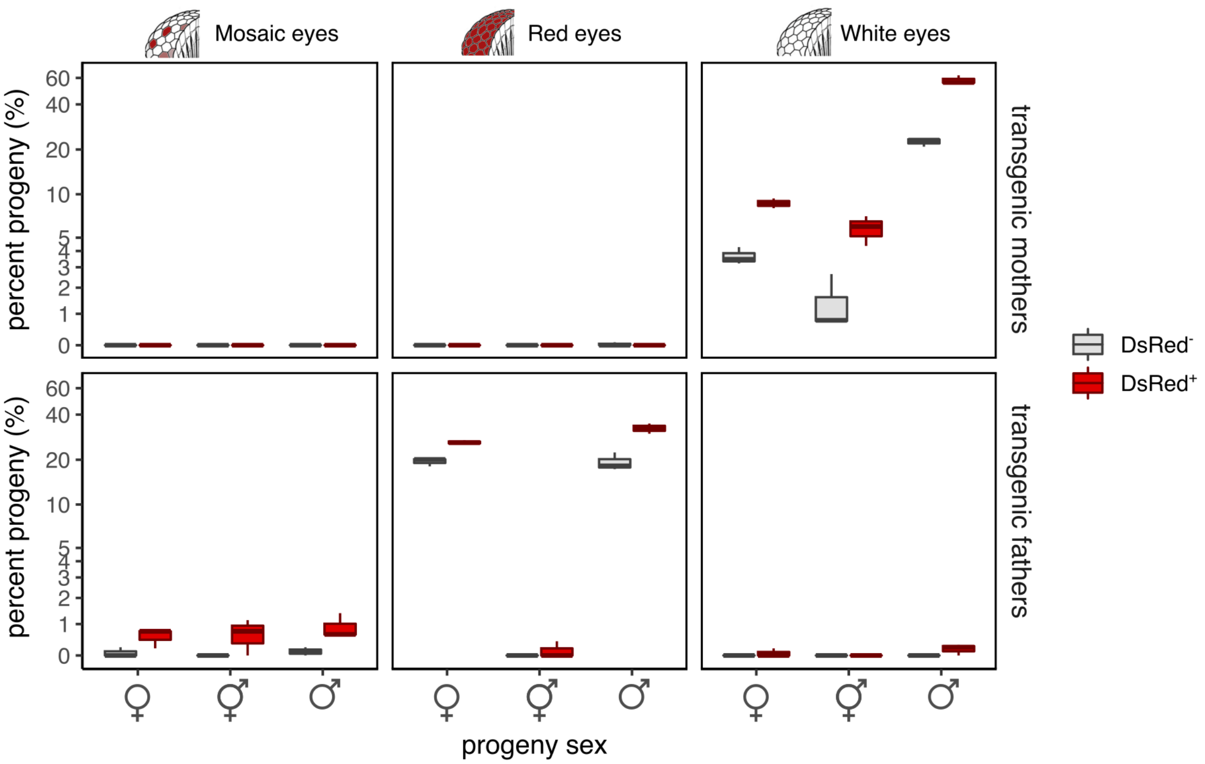

Figure S4. Eye pigmentation phenotypes in the offspring of *white-eye + tra* drive individuals. Eye pigmentation phenotypes of the DsRed positive and DsRed negative fractions of the progeny of transgenic fathers or mothers carrying the *white-eye + tra* drive constructs. Shown is the percentage of red eyed, mosaic and white eyed progeny obtained. Source data are provided as a Source Data file.

Figure S5.

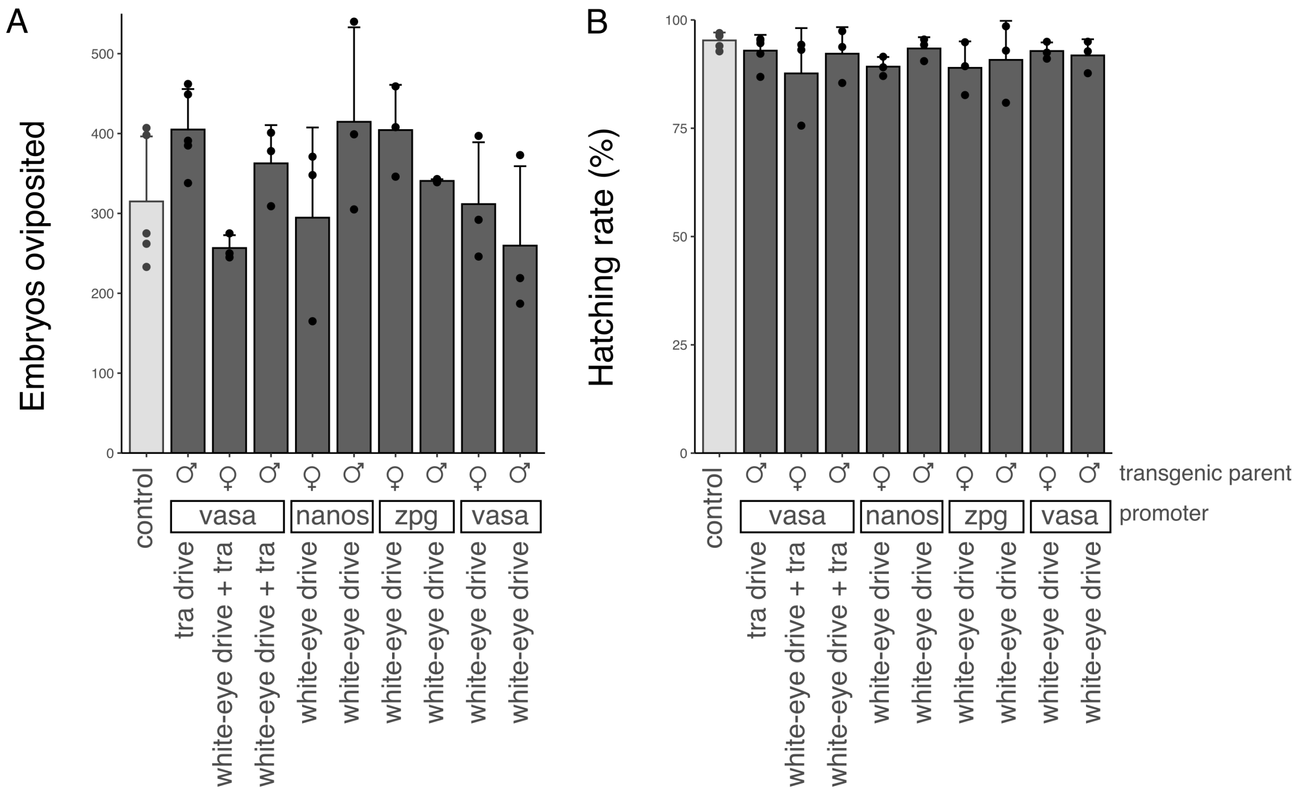

Figure S5. Fertility and fecundity of transgenic medfly strains. A. Average egg output of pooled crosses (10 males x 20 females) of hemizygous transgenic medflies when crossed to wild type individuals compared to wild type control intercrosses. **B.** The larval hatching rate of the progeny of hemizygous transgenic and wild type individuals compared to wild type control intercrosses. Error bars indicate the standard deviation around the mean. The total number of embryos scored were n=1757 for the control, n=2025 for the tra drive, n=1858 for *white-eye + tra* drive and n=2128/2235/1714 (*nanos*/*vasa*/*zpg*) for the *white-eye* drive groups with a minimum of 3 replicate experiments performed per group. Source data are provided as a Source Data file.

Figure S6.

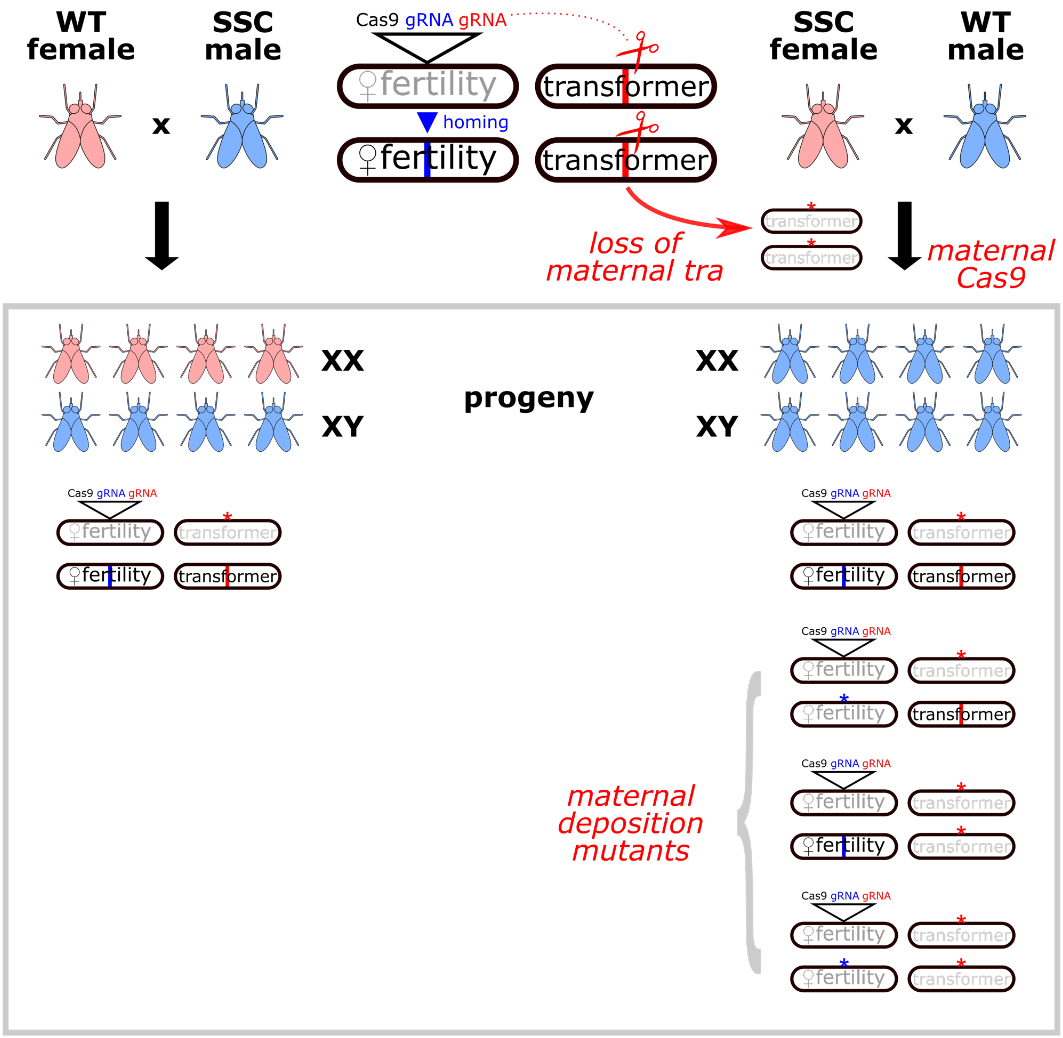

Figure S6. The sterilizing sex conversion (SSC) strategy. The SSC construct, designed to be active in both the male and female germline, homes into a female fertility gene while targeting the medfly transformer gene with a secondary gRNA. When SSC males are crossed to WT females (left cross) the resulting progeny, although inheriting the SSC construct preferentially due to gene drive, would be expected to show sex bias due to maternal rescue of transformer function. When SSC females are crossed to WT males (right cross) the maternal provision of transformer would be disrupted in a process that potentially acts dominantly. The resulting progeny, in addition to preferentially inheriting the SSC construct due to gene drive, would thus consist predominantly or exclusively of XX and XY males. Because of the loss of the maternal provision of transformer, potential resistance alleles generated by the maternal activity of Cas9 in the female fertility target gene and/or the transformer gene (maternal deposition mutants) would not be selected for in this generation of flies because they would be situated in phenotypic males. The grey box shows the possible progeny of these crosses and all mutant alleles that are the result of maternal activity and hence unavoidable even in a fully effective SSC construct. In all cases chromosomes inherited by the wild-type parent are shown on the bottom. Source data are provided as a Source Data file.

Supplementary Table 1

Marker	Sex	Fluorescence pattern	Tra genotype	Karyotype	Number
**DsRed positive**	Male	complete	mosaic	XY	41
				XX	8
		patchy	mosaic	XY	2
			mosaic*	XY	37
			WT	XY	3
	Intersex	complete	mosaic	XX	32
		patchy	mosaic	XX	3
			mosaic*	XX	39
**DsRed negative**	Male	N/A	WT / indel	XY	2
			WT / mosaic	XY	1
			WT / WT	XY	2

*WT trace visible

Supplementary Table 2

Parental cross to obtain males	Experimental cross	Male Karyotype	Transgenic progeny			Nontransgenic progeny			Total progeny	Transmission (%)
			Males	Females	Intersex	Male	Female	Intersex		
15 vasa drive ♂ x 30 wt ♀	1 vasa drive ♂ x 10 wt ♀	XY				n.d.				
	1 vasa drive ♂ x 10 wt ♀	XY				n.d.				
	1 vasa drive ♂ x 10 wt ♀	**XX**	0	0	91	0	10	29	130	0.70
	1 vasa drive ♂ x 10 wt ♀	XY				n.d.				
	1 vasa drive ♂ x 10 wt ♀	XY				n.d.				
	1 vasa drive ♂ x 10 wt ♀	XY				n.d.				
	1 vasa drive ♂ x 10 wt ♀	XY				n.d.				
	1 vasa drive ♂ x 10 wt ♀	XY				n.d.				
	1 vasa drive ♂ x 10 wt ♀	XY				n.d.				
	1 vasa drive ♂ x 10 wt ♀	**XX**	0	0	71	0	4	20	95	0.75
	1 vasa drive ♂ x 10 wt ♀	**XX**	0	0	110	0	10	27	147	0.75
	1 vasa drive ♂ x 10 wt ♀	XY				n.d.				
	1 vasa drive ♂ x 10 wt ♀	XY				n.d.				
	1 vasa drive ♂ x 10 wt ♀	XY				n.d.				
	1 vasa drive ♂ x 10 wt ♀	XY				n.d.				
	1 vasa drive ♂ x 10 wt ♀	XY				n.d.				
	1 vasa drive ♂ x 10 wt ♀	XY				n.d.				
	1 vasa drive ♂ x 10 wt ♀	XY				n.d.				
	1 vasa drive ♂ x 10 wt ♀	XY				n.d.				
	1 vasa drive ♂ x 10 wt ♀	XY				n.d.				
	1 non-transgenic ♂ x 10 wt ♀	XY				n.d.				
	1 non-transgenic ♂ x 10 wt ♀	XY				n.d.				
	1 non-transgenic ♂ x 10 wt ♀	XY				n.d.				
	1 non-transgenic ♂ x 10 wt ♀	**XX**	0	0	0	0	178	0	178	
	1 non-transgenic ♂ x 10 wt ♀	XY				n.d.				
	1 non-transgenic ♂ x 10 wt ♀	XY				n.d.				
	1 non-transgenic ♂ x 10 wt ♀	**XX**	0	0	0	0	134	0	134	
	1 non-transgenic ♂ x 10 wt ♀	XY				n.d.				
	1 non-transgenic ♂ x 10 wt ♀	XY				n.d.				
	1 non-transgenic ♂ x 10 wt ♀	XY				n.d.				

Supplementary Table 3

Parent 1	Sexual phenotype parent 1	Parent 2	Sexual Phenotype parent 2	Replicate	Embryos oviposited	Hatched embryos
tra drive	pseudo_male intersex	wt	wt female	1	253	0
tra drive	pseudo_male intersex	wt	wt female	2	291	0
tra drive	pseudo_male intersex	wt	wt female	3	190	0
tra drive	pseudo_male intersex	wt	wt female	4	270	0
tra drive	pseudo_male intersex	wt	wt female	5	185	0
white + tra drive	pseudo_male intersex	wt	wt female	1	222	0
white + tra drive	pseudo_male intersex	wt	wt female	2	239	0
white + tra drive	pseudo_male intersex	wt	wt female	3	175	0
white + tra drive	pseudo_male intersex	wt	wt female	4	198	0
white + tra drive	pseudo_male intersex	wt	wt female	5	215	0
wt	wt male	tra drive	pseudo_female intersex	1	0	0
wt	wt male	tra drive	pseudo_female intersex	2	0	0
wt	wt male	tra drive	pseudo_female intersex	3	0	0
wt	wt male	tra drive	pseudo_female intersex	4	0	0
wt	wt male	tra drive	pseudo_female intersex	5	0	0
wt	wt male	white + tra drive	pseudo_female intersex	1	0	0
wt	wt male	white + tra drive	pseudo_female intersex	2	0	0
wt	wt male	white + tra drive	pseudo_female intersex	3	0	0
wt	wt male	white + tra drive	pseudo_female intersex	4	0	0
wt	wt male	white + tra drive	pseudo_female intersex	5	0	0
wt	wt male	wt	wt female	1	298	286
wt	wt male	wt	wt female	2	282	263
wt	wt male	wt	wt female	3	255	245

Supplementary Table 4

Primer ID	sequence 5'.>3'	Plasmid
Fw-WHL	TCTATAAATTACGTTGGGCGGGA	
Rev-WHL	GATAAACCCTTCACCCGACCC	
Fw-WHR	GGGTGGCGAGCGTAAGC	
Rev-WHR	ACAGGCAGTTCGGTGGTGAA	
Gb-For-WHL	GAAACAGCTATGACCATGATTACGCCACTAGTCCGAGGCCTCTATAAATTACGTTGGGCGGGA	**Backbone pHA-white-dsRED**
Gb-Rev-WHL	GGAGAACTATATGGCGCGCCGATAAACCCTTCACCCGACCC	
Gb-For-WHR	ATGTATCTTAAGGCACGCGTGGGTGGCGAGCGTAAGC	
Gb-Rev-WHR	ATACGACTCACTATAGGGCGAATTGGGGATCGATCCACTAACAGGCAGTTCGGTGGTGAA	
Gb-For-U6-AscI	GGTCGGGTGAAGGGTTTATCGGCGCGCCATATAGTTCTCCCTACGATGACCTAT	
Gb-Rev-dsRED	GTCGCTTACGCTCGCCACCCACGCGTGCCTTAAGATACATTGATGAGTTTGG	
For-Prom-Nos	AGATCTTTACAATTTGTATTTCAGTTTA	
Rev-Prom-Nos	AATTATTACTACAGCTATCAGTATTACAA	
For-Term-Nos	GAAATTAGTTTTTTAAATTCTATCTTAATAGAAATTTC	
Rev-Term-Nos	ATGTTGGTAATGTCAATTACAAGAATG	
Gb-Fw-Prom-Nos	GGTCGGGTGAAGGGTTTATCAGATCTTTACAATTTGTATTTCAGTTTA	**pNanos-w**
Gb-Rev-Prom-Nos	CGTCGTGGTCCTTATAGTCCATAATTATTACTACAGCTATCAGTATTACA	
Gb-For-Term-Nos	GGCCGGCCAGGCAAAAAAGAAAAAGTAAGAAATTAGTTTTTTAAATTCTATCTTAATAGAAA	
Gb-Rev-Term-Nos	TCGATAGGTCATCGTAGGGAGAACTATATGGCGCGCCATGTTGGTAATGTCAATTACAA	
Gb-ForN-Cas9	GTAATACTGATAGCTGTAGTAATAATTATGGACTATAAGGACCACGACG	
Gb-RevN-Cas9	GAAATTTCTATTAAGATAGAATTTAAAAAACTAATTTCTTACTTTTTCTTTTTTGCCTGGCCGGCC	
For-Prom-Zpg	CTTTTGGGGGGCTCTTTTTTATT	
Rev-Prom-Zpg	ATTTATGGCTTGTGGAATGAATATTTCC	
For-Term-Zpg	TACAATTCATCGACTGACTTGTACAGATAC	
Rev-Term-Zpg	CCCGAAGCGAAATGCTGTCATTAATGTTG	
Gb-Fw-Prom-Zpg	GGTCGGGTGAAGGGTTTATCCTTTTGGGGGGCTCTTTTTTATT	**pZpg-w**
Gb-Rev-Prom-Zpg	TCCGTCGTGGTCCTTATAGTCCATATTTATGGCTTGTGGAATGAATATTTCC	
Gb-For-Term-Zpg	GGCCACGAAAAAGGCCGGCCAGGCAAAAAAGAAAAAGTAATACAATTCATCGACTGACTTGTACAGATACA	
Gb-Rev-Term-Zpg	CGATAGGTCATCGTAGGGAGAACTATATGGCGCGCCCCCGAAGCGAAATGCTGTCATTAATGTTG	
Gb-ForZ-Cas9	GGAAATATTCATTCCACAAGCCATAAATATGGACTATAAGGACCACGACGGAG	
Gb-RevZ-Cas9	GTATCTGTACAAGTCAGTCGATGAATTGTATTACTTTTTCTTTTTTGCCTGGCCGGCCTTTTTC	
For-Prom-Vasa	TTATAGCCTATTTCCACCAAGCAC	
Rev-Prom-Vasa	CATTTTCCGTGGTCTCTCTGAAAATAG	
For-Term-Vasa	TAGCACTACCACATCTATAAAAACAA	
Rev-Term-Vasa	CGCGCCGCAGTCGAGTCGTCATTTGTT	
Gb-Fw-Prom-Vasa	GGTCGGGTGAAGGGTTTATCTTATAGCCTATTTCCACCAAGC	**pVasa-w**
Gb-Rev-Prom-Vasa	AGTCTCCGTCGTGGTCCTTATAGTCCATTTTCCGTGGTCTCTCTGAAAATAGA	
Gb-For-Term-Vasa	GGCCAGGCAAAAAAGAAAAAGTAATAGCACTACCACATCTATAAAAACAATATAC	
Gb-Rev-Term-Vasa	TCGTAGGGAGAATATATGGCACAGGAAACAGCTATGACCGCGCCGCAGTCGAGTCGTCATTTGTT	
Gb-ForV-Cas9	CAGAGAGACCACGGAAAATGGACTATAAGGACCACGACGGAGAC	
Gb-RevV-Cas9	GTATATTGTTTTTATAGATGTGGTAGTGCTATTACTTTTTCTTTTTTGCCTGGCCGG	
Gb-ForU6-tra	TCGGTGCTTTTTTTTTTAATATATAGTTCTCCCTACGATGACCT	**pVasa-w-tra**
Gb-RevU6-tra	CGGCCTTGGCCGCGGTTAATAAAAAAAAAAGCACCGACTCGG	
Fw-traHL	GTCATAAACAAGTAGATGGTAAAATGTAC	
Rev-traHL	GATATCTCTACGTTTAATAACAACTTCGCTTG	
Fw-traHR	TTTGGTAATTTTAAAAGCATATTTTTTTCTTTG	
Rev-traHR	CCACAGGTGTCCCAGAGAA	
Gb-For-traHL	TACGCCACTAGTCCGAGGCCGTCATAAACAAGTAGATGGTAAAATGTAC	
Gb-Rev-traHL	ATGCTTTTAAAATTACCAAAGATATCTCTACGTTTAATAACAACTTCGC	
Gb-For-traHR	TTATTAAACGTAGAGATATCTTTGGTAATTTTAAAAGCATATTTTTTTCTTTG	
Gb-Rev-traHR	TGGGGATCGATCCACTAGTTCCACAGGTGTCCCAGAGAA	
Gb-For-U6-tra	TCGGTGCTTTTTTTTTTAATATATAGTTCTCCCTACGATGACCT	**pGdtra**
Gb-Rev-dsRED	CTTTTAAAATTACCAAAGATCCTTAAGATACATTGATGAGTTTGGA	
For-Prom-GdVasatra	GTTGTTATTAAACGTAGAGATGTTTTCCCAGTCACGACCGC	
Rev-Prom-GdVasatra	GGTCCTTATAGTCCATTTTCCGTG	
For-Term-GdVasatra	CGGCCACGAAAAAGGCCG	
Rev-Term-GdVasatra	GCTTTTAAAATTACCAAAGATCTCGAGACAGGAAACAGCTATGACCG	
Gb-ForV-Cas9	ACCAAACTTTAAATCTATTTTCAGAGAG	
Gb-RevV-Cas9	ATAGATGTGGTAGTGCTATTAC	

Supplementary Table 4

Primer ID	sequence 5'.>3'	Molecular analysis
F_Genome-w	CAGTTGCTCTTTCTACTCCGAAATTG	5' integration *white gene*
Rev_pNanos	TAGAACAATCTGAAGCTCTCCAATCG	
Rev_pZpg	ATTTATGGCTTGTGGAATGAATATTTCC	
Rev_pVasa	CGCAGAGAACGTAAATACATCGT	
For_dsRed	GGAGCGCGTGATGAACTT	3' integration white gene
F-w-tra	CATCCTACACAACACTGTGAAG	
Rev_Genome-w	GAAACATTGGCTACTAAGGTGACA	
F_Genome-tra	TTCTGGTAGCTGCCGTATGT	5' integration *tra gene*
For_Pub	CATGCGTTTCAGCTGTGTAT	3'integration tra gene
Rev_Genome-tra	CGGAAGACTATGCATCAAAATCC	
F-indel-w	ACGGCATATGACACAAAAACAG	Indel dection *white gene*
R-indel-w	TTCTGCGATAGCTTTTTCAACA	
F-indel-tra	CGAATTGAACAAAATGTACCCA	Indel dection tra *gene*
Rev-indel-tra	GAAACGGCATAAAGTGGTTC	
F-indel-w		genotype *white gene*
For_dsRed		
Rev_Genome-w		
For_Pub		genotype *tra gene*
F-indel-tra		
Rev_tra-screen	GAAACGGCATAAAGTGGTTC	
CcYF	GCTCGAAGACATGCATTGAA	karyotyping
CcYR	GACGGTAAGTGCCATTCGTT	
