# Supplementary material for: Gene drive and genetic sex conversion in the global agricultural pest Ceratitis capitata
Source: Nat Commun. 2024 Jan 8;15:372. doi: 10.1038/s41467-023-44399-1 (PMC10774415; doi:10.1038/s41467-023-44399-1)
Supplement: Supplementary file 2 — Reporting Summary [file 41467_2023_44399_MOESM2_ESM.pdf]

nature portfolio

Corresponding author(s): Dr Angela Meccariello and Dr Nikolai Windbichler

Last updated by author(s): 27-11-2023

Reporting Summary

Nature Portfolio wishes to improve the reproducibility of the work that we publish. This form provides structure for consistency and transparency in reporting. For further information on Nature Portfolio policies, see our Editorial Policies and the Editorial Policy Checklist.

Statistics

For all statistical analyses, confirm that the following items are present in the figure legend, table legend, main text, or Methods section.

n/a	Confirmed	
☐	☒	The exact sample size (*n*) for each experimental group/condition, given as a discrete number and unit of measurement
☒	☐	A statement on whether measurements were taken from distinct samples or whether the same sample was measured repeatedly
☐	☒	The statistical test(s) used AND whether they are one- or two-sided<br>*Only common tests should be described solely by name; describe more complex techniques in the Methods section.*
☒	☐	A description of all covariates tested
☒	☐	A description of any assumptions or corrections, such as tests of normality and adjustment for multiple comparisons
☐	☒	A full description of the statistical parameters including central tendency (e.g. means) or other basic estimates (e.g. regression coefficient) AND variation (e.g. standard deviation) or associated estimates of uncertainty (e.g. confidence intervals)
☐	☒	For null hypothesis testing, the test statistic (e.g. *F*, *t*, *r*) with confidence intervals, effect sizes, degrees of freedom and *P* value noted<br>*Give P values as exact values whenever suitable.*
☒	☐	For Bayesian analysis, information on the choice of priors and Markov chain Monte Carlo settings
☒	☐	For hierarchical and complex designs, identification of the appropriate level for tests and full reporting of outcomes
☒	☐	Estimates of effect sizes (e.g. Cohen's *d*, Pearson's *r*), indicating how they were calculated

Our web collection on statistics for biologists contains articles on many of the points above.

Software and code

Policy information about availability of computer code

Data collection	no software was used
Data analysis	R 4.2.3, https://github.com/genome-traffic/SuperMendelianSandbox , https://github.com/genome-traffic/gRandTheftAutosome

For manuscripts utilizing custom algorithms or software that are central to the research but not yet described in published literature, software must be made available to editors and reviewers. We strongly encourage code deposition in a community repository (e.g. GitHub). See the Nature Portfolio guidelines for submitting code & software for further information.

Data

Policy information about availability of data

All manuscripts must include a data availability statement. This statement should provide the following information, where applicable:

- Accession codes, unique identifiers, or web links for publicly available datasets
- A description of any restrictions on data availability
- For clinical datasets or third party data, please ensure that the statement adheres to our policy

All data needed to evaluate the conclusions in the paper are present in the paper and the Supplementary Materials. Input data and scripts to generate the figure panels presented in the paper available at https://github.com/genome-traffic/medfly_genedrive_paper. The SMS model code and parameters are available at https://github.com/genome-traffic/SuperMendelianSandbox and R scripts for visualisation are availaboe at https://github.com/genome-traffic/

gRandTheftAutosome. We utilized the Ccap2.1 reference genome assembly, the EGII-3.2.1 genome assembly as well as the Ccap2.1 reference annotation available via Genbank accessions GCA_000347755.4, GCA_905071925.1 and GCF_000347755.3 respectively.

Research involving human participants, their data, or biological material

Policy information about studies with human participants or human data. See also policy information about sex, gender (identity/presentation), and sexual orientation and race, ethnicity and racism.

Reporting on sex and gender	no human participants or human data was involved in this study
Reporting on race, ethnicity, or other socially relevant groupings	no human participants or human data was involved in this study
Population characteristics	no human participants or human data was involved in this study
Recruitment	no human participants or human data was involved in this study
Ethics oversight	no human participants or human data was involved in this study

Note that full information on the approval of the study protocol must also be provided in the manuscript.

Field-specific reporting

Please select the one below that is the best fit for your research. If you are not sure, read the appropriate sections before making your selection.

☒ Life sciences ☐ Behavioural & social sciences ☐ Ecological, evolutionary & environmental sciences

For a reference copy of the document with all sections, see nature.com/documents/nr-reporting-summary-flat.pdf

Life sciences study design

All studies must disclose on these points even when the disclosure is negative.

Sample size	In general insect gene drive studies are hugely overpowered compared to studies in e.g. mice or plants. This is due to the large number of progeny that can be obtained from even individual females. As stated in the text , clearly laid out in the figure legends, and as can be seen in the raw data tables we have counted thousands of individuals for every experiment and on top of that we are looking to identify effects of large sizes.
Data exclusions	No data was exluded from the analysis
Replication	This information is clearly stated in the figure legends and material and methods as well as in the "replicate" column of the raw data tables. The main gene drive experiments were done in either 3,5 or 10 replicates of pooled crosses with >10 individuals.
Randomization	Samples were selected at random through blind picking from groups separated through scoring using strict criteria (e.g. fluorescence, phenotypic eye colour and sex dimorphism) which included qualitative (e.g. representative images) and quantitative aspects (e.g. fly counts, sequencing results, electrophoresis)
Blinding	No formal blinding with another team member was conducted, however when counting or assessing phenotype classes from various numbered cages the parental genotypes were not immediately known to the experimenter. This is also a form of blinding that reduces detection and performance bias.

Reporting for specific materials, systems and methods

We require information from authors about some types of materials, experimental systems and methods used in many studies. Here, indicate whether each material, system or method listed is relevant to your study. If you are not sure if a list item applies to your research, read the appropriate section before selecting a response.

Materials & experimental systems

n/a	Involved in the study
☒	☐ Antibodies
☒	☐ Eukaryotic cell lines
☒	☐ Palaeontology and archaeology
☐	☒ Animals and other organisms
☒	☐ Clinical data
☒	☐ Dual use research of concern
☒	☐ Plants

Methods

n/a	Involved in the study
☒	☐ ChIP-seq
☒	☐ Flow cytometry
☒	☐ MRI-based neuroimaging

Animals and other research organisms

Policy information about studies involving animals; ARRIVE guidelines recommended for reporting animal research, and Sex and Gender in Research

Laboratory animals	Ceratitis capitata (Benakeion strain) INSECT
Wild animals	No wild animals were used in this study
Reporting on sex	Sex-based analysis was performed when sex-dependent experiments were carried out. Sex was assigned under the microscope via focusing on secondary sex characteristics of the medflies. Sex data is specified in the relevant results. The additional phenotype of intersex was assigned when a mixture of male and female secondary sex characteristics
Field-collected samples	no
Ethics oversight	No ethical approval was necessary as the organism used in this study is an insect and does not meet the criteria

Note that full information on the approval of the study protocol must also be provided in the manuscript.

Plants

Seed stocks	*Report on the source of all seed stocks or other plant material used. If applicable, state the seed stock centre and catalogue number. If plant specimens were collected from the field, describe the collection location, date and sampling procedures.*
Novel plant genotypes	*Describe the methods by which all novel plant genotypes were produced. This includes those generated by transgenic approaches, gene editing, chemical/radiation-based mutagenesis and hybridization. For transgenic lines, describe the transformation method, the number of independent lines analyzed and the generation upon which experiments were performed. For gene-edited lines, describe the editor used, the endogenous sequence targeted for editing, the targeting guide RNA sequence (if applicable) and how the editor was applied.*
Authentication	*Describe any authentication procedures for each seed stock used or novel genotype generated. Describe any experiments used to assess the effect of a mutation and, where applicable, how potential secondary effects (e.g. second site T-DNA insertions, mosiacism, off-target gene editing) were examined.*
